# The pupil-size artefact (PSA) across time, viewing direction, and different eye trackers

**DOI:** 10.3758/s13428-020-01512-2

**Published:** 2021-03-11

**Authors:** Ignace T. C. Hooge, Diederick C. Niehorster, Roy S. Hessels, Dixon Cleveland, Marcus Nyström

**Affiliations:** 1 Experimental Psychology, Helmholtz Institute, Utrecht University, Utrecht, The Netherlands; 2 Lund University Humanities Lab and Department of Psychology, Lund, Sweden; 3 Eyegaze Inc., Fairfax, VA USA; 4 Lund University Humanities Lab, Lund, Sweden

**Keywords:** Pupil-size artefact, Eye tracking, Accuracy, Gaze estimation, Monocular

## Abstract

The pupil size artefact (PSA) is the gaze deviation reported by an eye tracker during pupil size changes if the eye does not rotate. In the present study, we ask three questions: 1) how stable is the PSA over time, 2) does the PSA depend on properties of the eye tracker set up, and 3) does the PSA depend on the participants’ viewing direction? We found that the PSA is very stable over time for periods as long as 1 year, but may differ between participants. When comparing the magnitude of the PSA between eye trackers, we found the magnitude of the obtained PSA to be related to the direction of the eye-tracker-camera axis, suggesting that the angle between the participants’ viewing direction and the camera axis affects the PSA. We then investigated the PSA as a function of the participants’ viewing direction. The PSA was non-zero for viewing direction 0^∘^ and depended on the viewing direction. These findings corroborate the suggestion by Choe et al. (Vision Research **118**(6755):48–59, 2016), that the PSA can be described by an idiosyncratic and a viewing direction-dependent component. Based on a simulation, we cannot claim that the viewing direction-dependent component of the PSA is caused by the optics of the cornea.

## Introduction

Eye tracking is used in many branches of psychology, cognitive science, and medicine to investigate e.g., attention, perception, development of the oculomotor system, usability and to diagnose diseases associated with neurological disorders. Nowadays, the majority of eye trackers are non-invasive and use high-speed cameras to film the eye(s) illuminated with infrared light. In the pupil-minus-CR eye tracker (Merchant et al., 1974), the pupil center and the corneal reflection (CR) are used to estimate gaze (see Morimoto et al., 2000). The pupil and CR centers can be estimated by an algorithm calculating the center of mass (e.g. Mulligan, 1997) or by an ellipse fit (e.g., Kristek, 1965). One hidden assumption underlying the pupil-minus-CR technique is that the pupil center location is fixed with respect to the eyeball. Research showed that this assumption can be violated (Walsh, 1988). Wyatt (1995) investigated the form of the human pupil and showed that when the pupil changes size, the shape of the pupil also changes. Depending on the pupil-shape changes, the center of the pupil may shift. Wyatt (1995) predicted that if during fixation the pupil changes size and shape (relative to the size and the shape during calibration), the eye tracker will report a deviation from the computed gaze direction even if the eye did not actually rotate. To be able to observe and quantify this phenomenon, Wyatt (1995) built a setup consisting of a camera, a half-silvered mirror, and a photographic slit lamp to produce bright retinal reflections (the red eye effect). In 1995, Wyatt warned that his finding had implications for gaze estimation. Indeed in 2010, Wyatt showed erroneous deviations of computed gaze with changing pupil size using an eye tracker (ISCAN EC-101 60 Hz; dark pupil technique). The average deviation in seven participants was 0.81^∘^ with a maximum deviation of 1.22^∘^. From here on, we will refer to this erroneous deviation of computed gaze as the pupil-size artefact (PSA).

Since Wyatt’s description of the pupil-size artefact, several researchers have investigated this artefact. Wildenmann and Schaeffel (2013) tested the prediction of Wyatt (1995) directly with a self-built eye tracker (in contrast to a corporate eye tracker as used by Wyatt, which is basically a black box to the end user). They filmed the eye frontally from below with an IR camera (640 × 480 pixels @87 Hz) while they illuminated the eye with an IR-led. The recorded eye images were processed by various computer vision techniques to determine pupil size, location of the pupil center, and gaze direction. They replicated the pupil-size artefact and showed a direct relation between pupil size and pupil-center locations in an experimental setting in which they were in control of the algorithms used to compute these values. Wildenmann and Schaeffel (2013) also reported that the pupil-size artefact is similar across the eyes in magnitude and direction, but mirrored in the right eye with respect to the left eye. Drewes et al., (2014) replicated the pupil-size artefact in a high-end eye tracker (SR Research EyeLink 1000) in a much higher number of participants (*n* = 39) than Wyatt (2010, *n* = 7) and Wildenmann and Schaeffel(2013, *n* = 10). Drewes et al., (2014) report much larger deviations (up to 5^∘^) and in contrast to Wildenmann and Schaeffel (2013), they report large differences between the magnitude of the pupil-size artefact obtained in the different eyes (0.1^∘^ to 3.0^∘^, mean difference 1.0^∘^). Hooge et al., (2019) claim that the pupil-size artefact in the separate eyes causes video eye trackers to be too inaccurate to report valid binocular eye-tracking measures for the larger fixation distances (about 50 cm and further). They were also the first to show that the eyes did actually not rotate during the pupil-size artefact. To do so, they separately investigated the pupil and CR signals during fixation while the pupil size changed due to changing lighting conditions; the pupil center signals showed apparent movement whereas the CR signals did not show signs of eyeball rotation.

Besides description of the nature of the pupil-size artefact, researchers’ efforts have mostly focused on compensation methods for the pupil-size artefact. In retrospect, Merchant et al., (1974) already mentioned the pupil-size artefact in some participants without knowing the cause. They also presented a method to compensate for it. On page 314 they wrote:

“In this case, changes in pupil diameter will produce an apparent, but erroneous, change of eye direction. To compensate for this effect, the null vector (*X*_0_,*Y*_0_) is stored not as a single quantity, but as a table of 64 values for each of 64 different, values of pupil diameter”.

Wyatt (2010) also proposed a compensation method that reduces the erroneous deviation up to 45% of its original magnitude.

Based on the suppositions that a) a significant portion of an eye’s physiological pupil-center shift results from repeatable performance of the pupillary muscles during dilation and constriction, and b) that such muscle behaviors may vary significantly from eye to eye, Cleveland (2003) formulated a calibration method that utilizes varying scene lighting to stimulate dilation/constriction and constructs a pupil-shift curve as a function of pupil diameter. As suggested by Merchant, the eye tracker shifts the apparent pupil center during runtime in accordance with the calibrated pupil-shift curve. No formal experiments have been run to quantify the effects of the Cleveland approach.

Drewes et al., (2014) designed and validated various methods to compensate for the pupil-size artefact. Their methods involve offline calibration under different lighting conditions to evoke large and small pupils. Drewes et al., (2014) reported up to 74% compensation for the pupil-size artefact. The artefact may deteriorate gaze measurements in many situations where pupil size changes. For example, the pupil size may change over the span of an experiment due to arousal and cognitive processing. To compensate for the arousal related pupil-size artefact, Choe et al., (2016) presented an offline compensation method to regress out pupil size changes during fixation, which increases accuracy of the eye-tracking data. Another cause of pupil size change is the near triad or accommodation reflex. Together with accommodation and convergence, both pupils constrict when a nearby object is fixated binocularly. Jaschinski (2016) investigated the pupil-size artefact during fixation of targets at near distances (24, 30, and 40 cm). He described the pupil-size artefact in the binocular domain for nearby targets. He also proposed a compensation method for the binocular near distance pupil-size artefact.

The pupil is not a physical entity, it is a hole in the iris. The iris has been compared with a curtain in a bathtub full of water (J. van der Steen, personal communication). This beckons the question about stability over time of the shape-size relations. Wyatt (1995) measured pupil shapes of four participants on a short and on a longer time scale. The main result was that: “Shapes were usually stable within a session and could remain fairly stable for at least a year”. Wyatt (1995) concludes: “The data do not permit an assessment of the time course of changes—closely separated sessions could be as different as widely separated ones”. It seems that the pupil-shape pupil-size relation is a stable one.

In the present study, we are interested in the stability over time of the pupil-size artefact as measured with an eye tracker. We aim to replicate the findings of Wyatt (1995) for the pupil-size artefact by investigating the long-term stability.

We do not know a lot about the pupil-size artefact. We wonder whether the pupil-size artefact is related to 1) the measurement device, 2) the algorithm inside the eye tracker, 3) the geometry of the whole setup^1^, 4) the eye physiology. We will present a series of six experiments conducted with the EyeLink 1000 Plus, the Tobii Pro Spectrum and our self-built eye tracker which we will refer to as FLEX. We incorporate FLEX because most corporate eye trackers are black boxes to the end user with respect to the filtering and algorithms used to compute gaze from the extracted pupil center and corneal reflection(s). In this article, we will answer questions about the stability of the PSA over time and whether the PSA depends on eye-tracker geometry and viewing direction.

## Experiment 1. Short-term stability of the pupil-size artefact

Based on measurements done with a camera, Wyatt (1995) reported that the relation between the pupil size and the pupil shape is stable. We are interested in whether this is also true for the pupil-size artefact (i.e., the deviation in gaze reported by an eye tracker due to the pupil-size changes). In Experiment 1, we tested the stability of the monocular pupil-size artefact by measuring it ten times in a row for two participants.

### Methods

#### Setup

Binocular eye movements were recorded with an SR Research EyeLink 1000 Plus (host software v. 5.12) at 1000 Hz (pupil area mode; heuristic filters turned off; pupil detection model was center of mass; default binocular nine-point EyeLink calibration with nine-point validation; grey background). To minimize head movements, we used the standard EyeLink 1000 Plus chin and forehead rest. The visual stimulus was presented on a 24-inch EIZO FlexScan EV2451 (52.8 × 29.7 cm; 1920 pixels × 1080 pixels; 16:9 ratio; refresh rate: 60 Hz) placed at a distance of 77 cm from the eye. Stimulus presentation was done with PsychoPy v.1.83.01 (Peirce, 2007, 2008) and the EyeLink Dev. Kit (v.1.11.571) was used to communicate with the EyeLink Host computer. The light in the experimental room was turned off, however it was not completely dark because one of the walls contained a window to the corridor next to the lab, which was dimly lit. This setup is almost identical to the Lund setup used in Hooge et al., (2019).

#### Stimulus and task

The stimulus to evoke slow pupil size changes consisted of one fixation marker (a blue disk with a diameter of 0.6^∘^ with a red center dot with a diameter 0.1^∘^) placed on a background that slowly changed from black to white and back with a frequency of 0.125 Hz following a sinusoidal profile. Presentation time was 160 s (20 cycles of 8 s). The participants were asked to continuously fixate the marker without blinking during stimulus presentation. Each of the ten trials was preceded by a binocular nine-point EyeLink calibration with nine-point validation.

#### Participants

Two males, p1 (the first author) and p2 (the last author) were engaged in Experiment 1. Both p1 and p2 are experienced with eye-tracking experiments. Written informed consent was provided by the participants, and the experiment was conducted in accordance with the Code of Ethics of the World Medical Association (Declaration of Helsinki).

#### Signals

In this experiment, we use two types of signals. We use the horizontal and vertical components of the calibrated pupil-minus-CR signal (expressed in degrees) and we refer to this signal as the gaze signal. The second signal is the pupil diameter in mm. The original pupil-size signal of the EyeLink 1000 Plus is expressed in arbitrary units (SR Research, 2009). We converted the pupil size signal to millimeters by a ratio that was determined with an off-line calibration. The off-line calibration consisted of recording of a black circle placed at the position of the left eye to determine the conversion ratio. To enable off-line calibration we made sure that we recorded eye images with non-occluded pupils.

In the pupil signal, a blink may consist of a period of missing data flanked with episodes of fast pupil-size changes. We are only interested in the slow parts of the pupil signal that are related to the slow changes in the light level. To remove the fast parts, we first computed a pupil velocity signal with a filter based on the one described in Hooge and Camps (2013). Then we replaced all samples having a pupil-size velocity greater than two standard deviations above the average value by NaN (not a number). To ensure that all fast episodes were removed, we subsequently replaced 20 ms of data left and right from all periods of missing data by NaN.

### Results and discussion

**Fig. 1:**
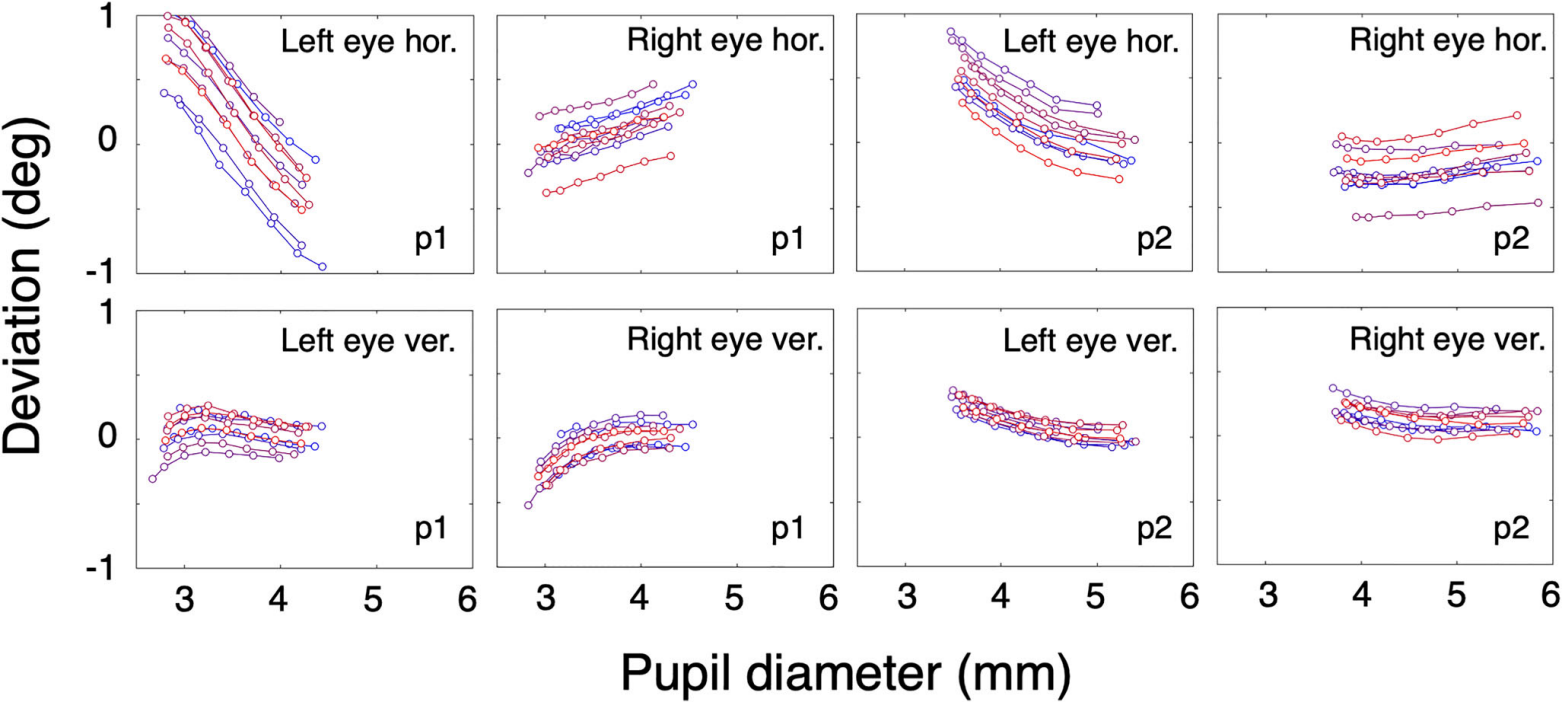
Short-term pupil-size artefact stability. The *left four panels* contain results of p1, the *right panels* contain results of p2. Different *lines* represent data from different trials. Each *line* consists of seven points, *x*-values representing the second until the eighth decile of pupil size with steps of one-tenth, *y*-values represent the mean of the corresponding deviation values. In this experiment, each measurement (one colored line in one of the panels) was preceded by a nine-point calibration (*grey background*)

Figure 1 shows horizontal and vertical components of the gaze signal as a function of pupil diameter while the participant fixates the center of the screen (0^∘^, 0^∘^). Each line represents gaze data from one trial of 160 s. The proportion of data loss (empty samples) per trial ranged from 0.000 to 0.117, with a mean value of 0.0543 and a standard deviation of 0.0479. The individual lines in one panel have similar shapes that reflect the relation between pupil size and deviation. However, the individual lines are separated in the vertical direction. For both participants, the pupil-size artefact is about similar in magnitude and comparable in shape for the vertical component in both eyes. The artefact in the horizontal component is different for the left and right eyes and seems to be bigger in the left eye.

**Fig. 2:**
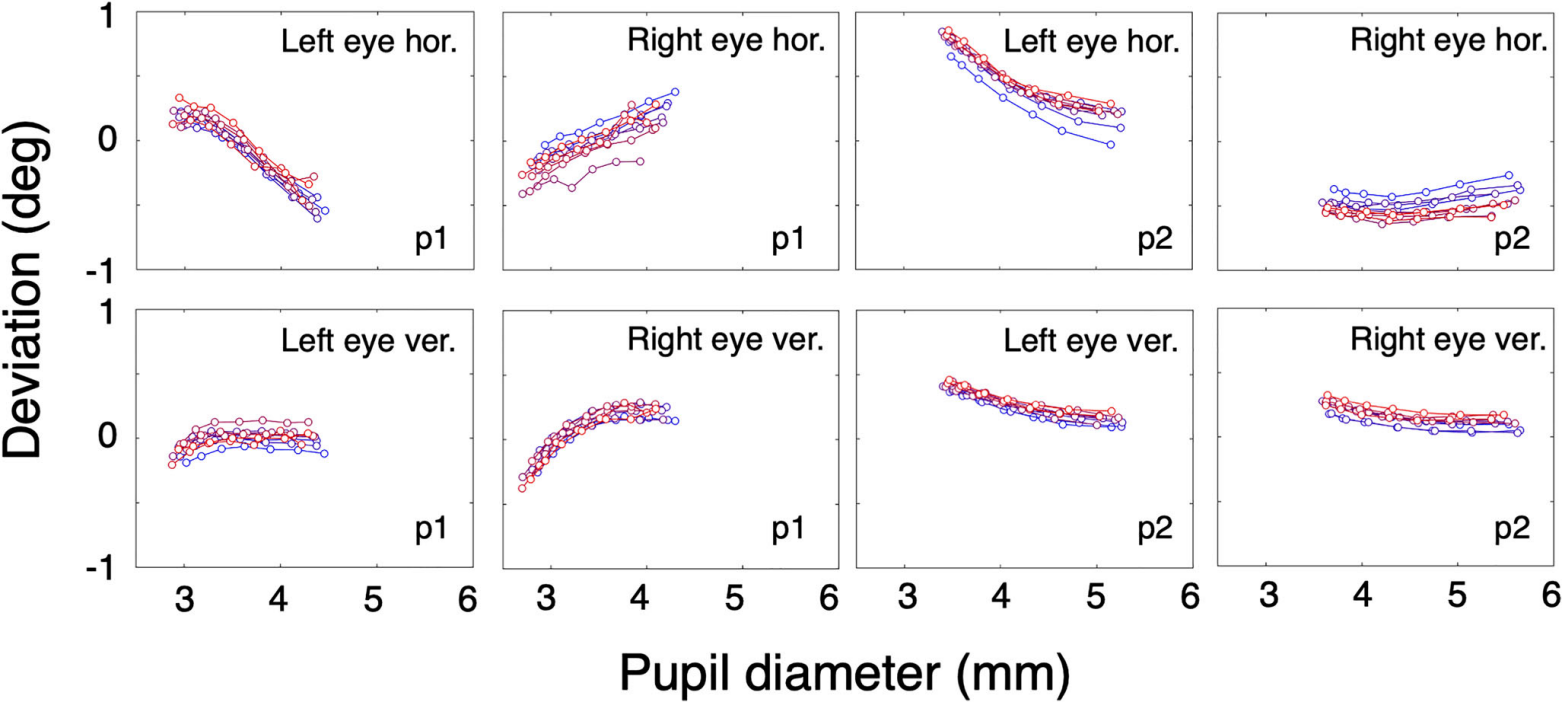
Short-term pupil-size artefact stability. The *left four panels* contain results of p1, the *right panels* contain results of p2. Individual *lines* in one panel represent data from different trials. Each *line* represents data from one trial and consists of seven points, *x*-values representing the second until the eighth decile of pupil size with steps of one-tenth, *y*-values represent the mean of the corresponding deviation values. In this experiment, all ten measurements were preceded by one nine-point calibration (*grey background*)

The variation between the individual measurements is reflected in the vertical offsets between the lines that are clearly visible in each panel. To exclude that these offsets are related to calibration errors, we repeated this experiment with the only difference that the eye tracker was only calibrated once preceding the first trial. We hypothesize that the inaccuracy due to recalibration is mostly due to the inability of the participants to exactly repeat fixation of the whole grid of calibration targets. Figure 2 contains the results of the experiment with only one calibration preceding the first trial. The proportion of data loss (empty samples) per trial ranged from 0.001 to 0.0292, with a mean value of 0.0117 and a standard deviation of 0.106. Comparison of the panels of Figs. 1 and 2 reveals that the curves describing the relation between the pupil size and deviation are similar. The difference between the figures are the vertical offsets that are clearly present in Fig. 1 and are much smaller in Fig. 2.

Experiment 1 (Figs. 1 and 2) shows that test–retest reliability of the pupil-size artefact on a short time scale (within 1 h) is very high. Both participants show idiosyncratic deviation patterns as a function of pupil size and both participants have a larger magnitude of the pupil-size artefact in the left eye. Some studies suggest that the pupil-center displacement is symmetric in the two eyes. Wildenmann and Schaeffel (2013) wrote: “The pupil center was also displaced superiorly although not with perfect symmetry in both eyes”, Jaschinski (2016) wrote: “Concerning the pupil center shift, it has been reported earlier that a reduction in pupil size typically shifts the center of the pupil nasally”. Drewes et al., (2014) reported that the direction of the pupil-size artefact in both eyes was in the nasal direction, however magnitudes of the pupil-size artefacts may differ between the eyes (0.1^∘^ to 3.0^∘^, mean difference 1.0^∘^).

A new calibration seems to introduce an offset with respect to the previously calibrated data (vertical offsets between the lines, see Fig. 1). The effect of a new calibration is comparable to a random shift of the gaze positions with a value varying from 0^∘^ to the accuracy of the eye tracker (in our case the manufacturer reports 0.5^∘^). In the present experiment, the deviation introduced by a recalibration is of the order of deviations caused by the pupil-size artefact. The signals from consecutive trials of the experiment without calibrations between the measurements, showed more similarity than the signals from the experiment with calibration for each trial (compare Fig. 2 to Fig. 1). We hypothesize that the inaccuracy due to recalibration is mostly due to the inability of the participants to exactly repeat fixation of the whole grid of calibration targets. From Experiment 1, we conclude that the pupil-size artefact is very reproducible on a time scale shorter than 1 h. In the next experiment, we test the reliability of the pupil artefact on a longer time scale (days).

## Experiment 2. Stability of the pupil-size artefact on a time scale of days

Within a period of 11 days (on days 1, 2, 3, 4, 5, 10, and 11, respectively), participants p1 and p2 each performed one measurement per day. The setup of Experiment 2 is similar to the setup of Experiment 1. Each trial was preceded by a standard nine-point calibration (grey background).

### Results and discussion

**Fig. 3:**
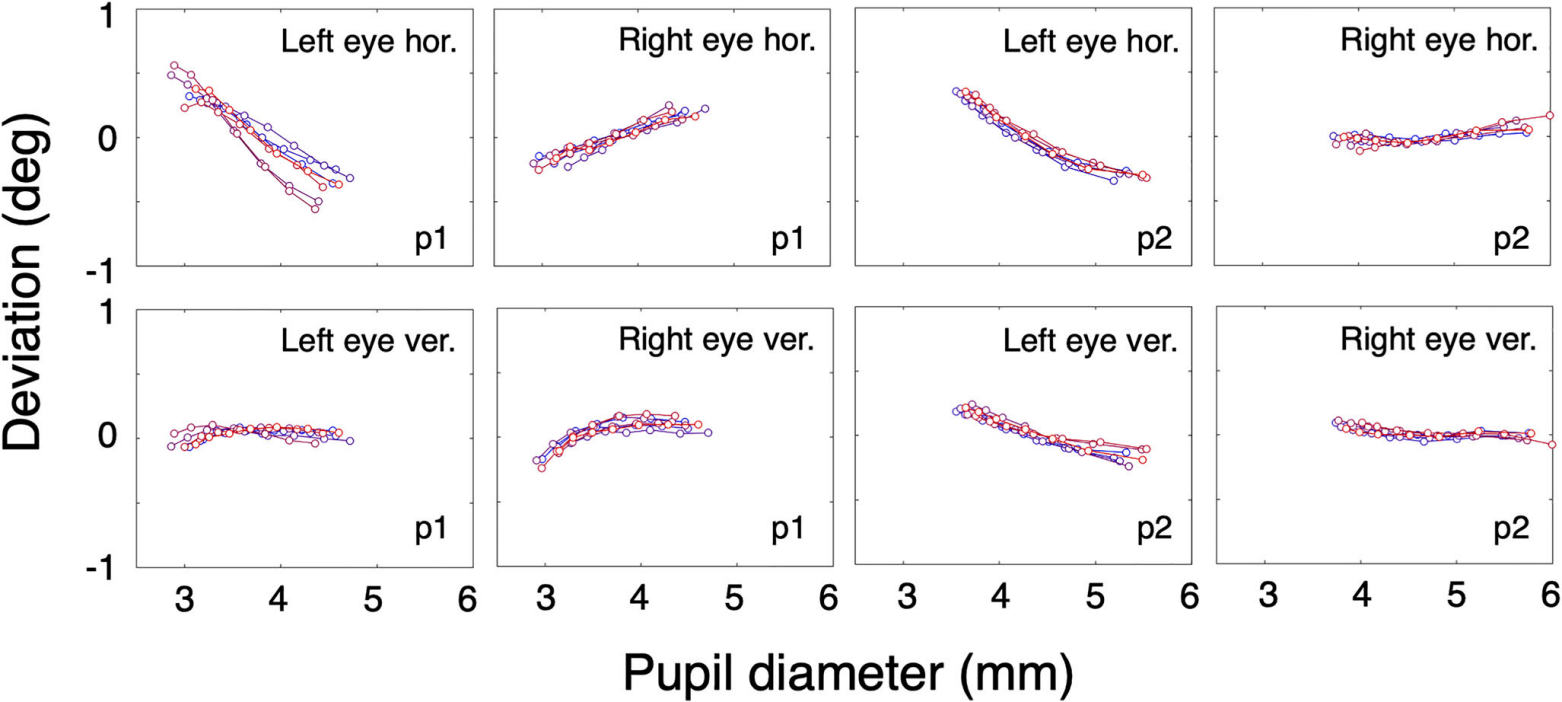
Mid-term pupil-size artefact stability. The *left four panels* contain results of p1, the *right panels* contain results of p2. Each *line* represents data from one trial and consists of seven points, *x*-values representing the second until the eighth decile of pupil size with steps of one-tenth, *y*-values represent the mean of the corresponding deviation values. In this experiment, each measurement (represented by one colored line in one of the panels) was preceded by a nine-point calibration (*grey background*). Each measurement took place on a different day

The proportion of data loss (empty samples) per trial ranged from 0.001 to 0.115, with a mean value of 0.0576 and a standard deviation of 0.0452. Because we are mainly interested in the gaze deviation change as a function of pupil size, we subtracted the median deviation from the signal of each trial. Figure 3 shows the pupil-size artefact over a period of 11 days. The artefact seems to be stable and not very different from the artefacts shown in Figs. 1 and 2.

## Experiment 3. Long-term stability of the pupil-size artefact

To investigate the long-term stability, we compared measurements from participants in Hooge et al., (2019) conducted in the spring 2018 with new measurements in the same participants (conducted in the spring of 2019). We used two set ups (one in Utrecht, the Netherlands, the other in Lund, Sweden). In Utrecht, we used a Samsung 2433BW monitor (refresh rate of 60 Hz) and an EyeLink 1000 (at 500 Hz). In Lund, we used an ASUS VG248QE monitor (refresh rate 240 Hz) and an EyeLink 1000 Plus (at 1000 Hz).

### Result and discussion

**Fig. 4:**
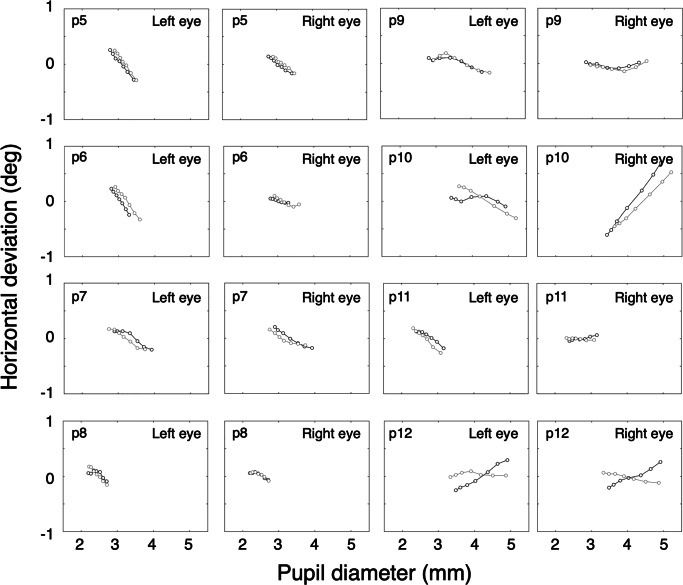
Long-term pupil-size artefact stability. *Black* denotes the measurement done in the spring of 2018. *Grey* denotes the measurement done in the spring of 2019. Each *line* represents data from one trial and consists of seven points, *x*-values representing the second until the eighth decile of pupil size with steps of one- tenth, *y*-values represent the mean of the corresponding deviation values

The results are depicted in Fig. 4. The proportion of data loss (empty samples) per trial ranged from 0.076 to 0.106, with a mean value of 0.087 and a standard deviation of 0.0135. Seven out of eight participants show a pupil-size artefact that is comparable when measured twice with a pause of about 1 year in between. For one participant (p12), the PSAs are very different. Currently, we have no explanation for this.

## Experiment 4. Is the pupil-size artefact eye-tracker specific?

Since 2010, the pupil-size artefact has been studied with a few different eye trackers (Wyatt, 2010; Drewes et al., 2012, 2014; Choe et al., 2016; Jaschinski, 2016; Hooge et al., 2019) and with a self-built camera system (Wildenmann and Schaeffel, 2013). Wyatt (2010) (ISCAN EC-101) and Wildenmann and Schaeffel (2013) (self-built eye tracker) describe the pupil-size artefact as symmetric. This means that the pupil-size artefact is similar in the left and right eyes but mirrored. In contrast, Drewes et al., (2014) (SR Research EyeLink 1000) reported that the PSA between the left and the right eyes differs. Drewes et al., (2014) wrote: “In many participants, large differences in the drift magnitude were found between the left and the right eye; for this analysis, the left and right eyes of each participant were therefore treated as independent samples”. Two other studies (Hooge et al., 2019; Choe et al., 2016) also used an EyeLink 1000. Hooge et al., (2019) reported only binocular (vergence) data. Choe et al., (2016) also reported differences of the PSA between left and right eyes. We therefore wonder whether differences between studies might be attributed to the use of different eye trackers. As such, we investigate whether there is an eye tracker-dependent pupil-size artefact. To investigate this question, we repeated our experiment with a Tobii Pro Spectrum (an eye tracker that has not yet been used to investigate the pupil-size artefact). From here on, we refer to the Tobii Pro Spectrum as Spectrum and to the EyeLinks as the EL1000 and EL1000p (plus).

### Setup and participants

The setup is identical to the setup used in Experiments 1 and 2, except that we used the Spectrum (firmware version 1.7.6.) instead of the EL1000p. We engaged four participants in Experiment 4 (including p1 and p2 from Experiments 1 to 3). Participant p3 is the second author of this article and p4 is a male volunteer who is not naive with respect to the goals of this experiment.

### Results and discussion

**Fig. 5:**
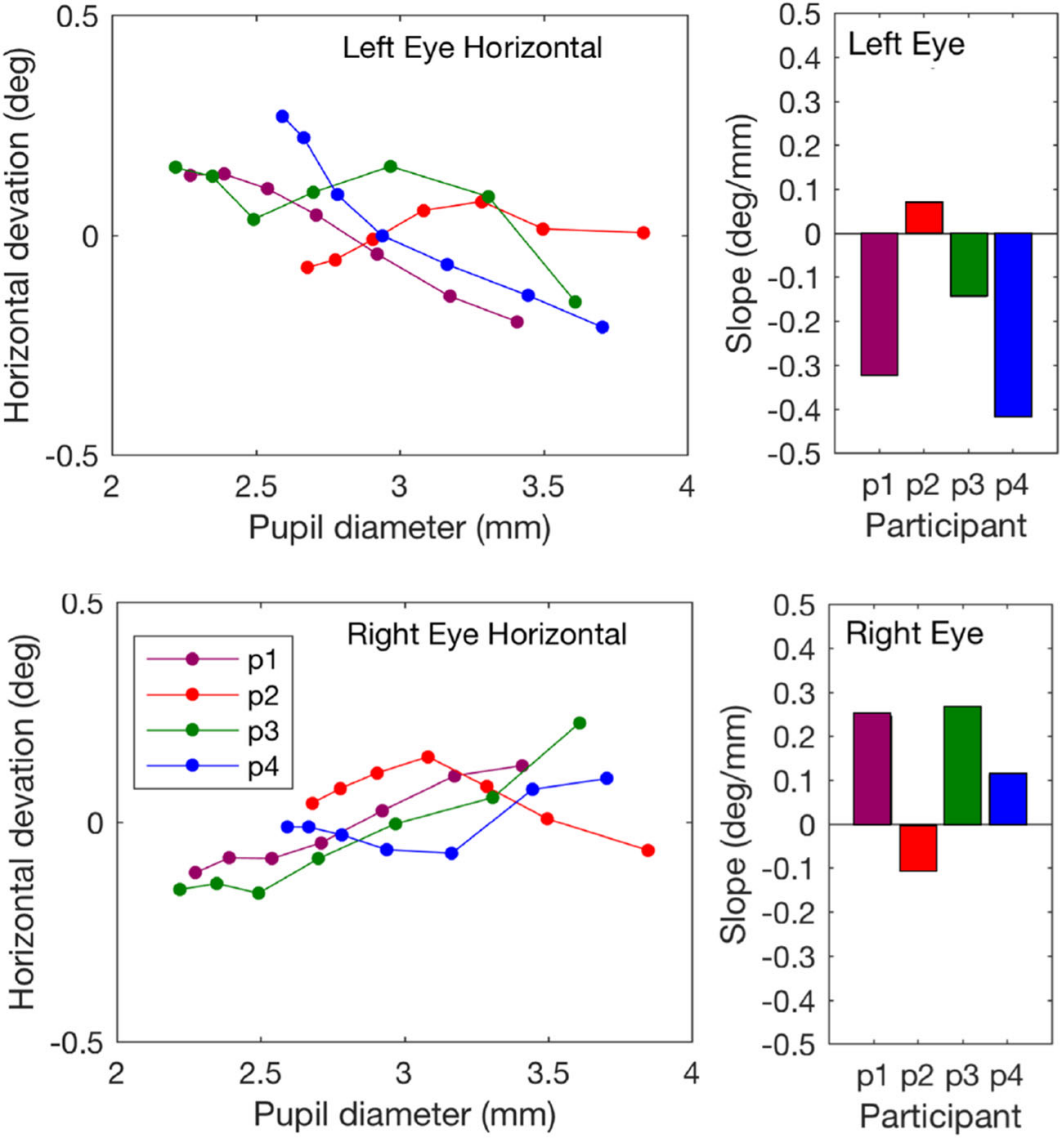
The pupil-size artefact in the Spectrum. The horizontal component of the pupil-size artefact in the left and right eyes for four participants. Each *line* represents data from one trial and consists of seven points, *x*-values representing the second until the eighth decile of pupil size with steps of one-tenth, *y*-values represent the mean of the corresponding deviation values. Participants p1 and p2 took part in Experiment 1-3. The artefact is symmetric in magnitude for both the left and the right eye (for perfect symmetry, slopes in the left and the right eye should have similar magnitudes and opposite signs). The magnitude of the pupil-size artefact in the Spectrum is also smaller than for the EyeLink 1000

Figure 5 shows the horizontal component of the pupil-size artefact in the left and right eyes measured with the Spectrum. The proportion of data loss (empty samples) per trial ranged from 0.001 to 0.120, with a mean value of 0.0585 and a standard deviation of 0.0529. The difference from the asymmetric EL1000p data is clear (see Figs. 1, 2 and 3). In the data from the Spectrum, the deviation in the left eye is about similar in size to that of the right eye, but mirrored. Participants p1, p3, and p4 have negative slopes for the right eye and positive slopes for the left eye. Participant p2 has a slightly positive slope for the left eye and a slightly negative slope for the right eye. The results for participants p1 and p2 are interesting because they both had negative slopes for the pupil-size artefact of the left eye when measured with the EyeLink. When measured with the Spectrum, the slopes for both the left and right eye pupil-size artefacts were shifted in the positive direction. In summary, the pupil-size artefact seemed asymmetrical in Experiments 1–3. These experiments have in common that the eye tracker used was the EL1000 or the EL1000p. The pupil-size artefact in the Spectrum was symmetric (i.e., slopes of the PSA in the two eyes had opposite signs) as opposed to the pupil-size artefact in the EL1000p.

Based on these results, we took a closer look at the differences between the geometry of the EyeLink setup and the Spectrum setup. When used in desktop mode right in front of the participant, the EyeLink setup is asymmetrical. The camera films the face of the participant from the left side (under 15^∘^, 13 cm to the left at a distance of 50 cm) while the IR-LED cluster illuminates the face from the right side (Choe et al., 2016, Fig. 1). The Spectrum eye tracker is symmetrical, it films both eyes with two cameras placed symmetrically and centrally below the monitor. The four IR illuminators are placed around the cameras (for bright pupil eye tracking) and left and right below the monitor (for dark pupil eye tracking). Besides the geometry there are other differences between the Spectrum and the EyeLink. Although there are other differences between the Spectrum and the EL1000p, we here concern ourselves only with the differences in geometry.

## Experiment 5. Is the pupil-size artefact asymmetric?

**Fig. 6:**
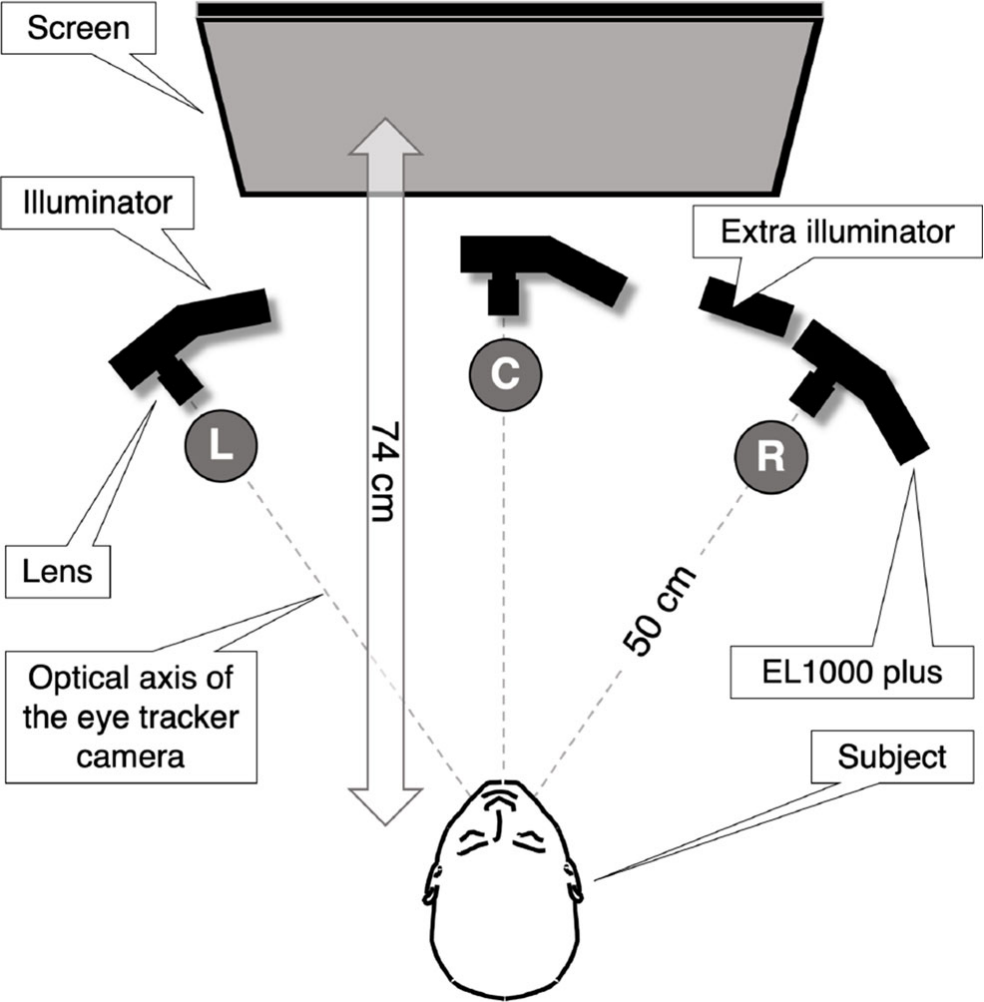
The setup used in Experiment 5. *L* depicts the left condition, *C* and *R*, respectively, the center and right condition. In the left and right conditions, the camera axis is rotated over − 18^∘^ and 18^∘^. In the left (*L*) and the center condition (*C*) we used the standard IR illuminator. In the right condition (*R*), we used a separate illuminator instead of the standard illuminator. This illuminator was placed on the left side of the eye-tracker camera and the standard illuminator was disabled

When measured with the EL1000p, the slope of the pupil-size artefact in the left eye is steeper than in the right eye. In the Spectrum data, we found a more symmetrical pupil-size artefact. One of the differences between the two eye trackers is the relative orientation of the camera(s) with respect to the head of the participant. This suggests that camera orientation may play a role in the relative magnitudes of the pupil-size artefacts in the left and the right eyes. To investigate this, we rotated our EL1000p. We filmed the eyes from the left side, the center and the right side (see Fig. 6). We expect the PSA of the center condition to resemble the PSA from the Spectrum more than the PSA from the left and right positions.

### Setup and participants

In the first trial, we rotated the EyeLink camera so that the optical axis of the camera pointed at a location between the two eyes (denoted with C in Fig. 6). In the other two trials, we respectively rotated the eye tracker camera 18^∘^ to the left (denoted with L in Fig. 6) and 18^∘^ to the right (denoted with R in Fig. 6) over a virtual circle (r = 50 cm; center located between the two eyes). When rotated to the left (L), the EyeLink camera was slightly further rotated (− 18^∘^ versus − 15^∘^) than in desktop configuration (see Choe et al., 2016, figure 1, p. 49). When rotated to the right (R), we used a separate IR-illuminator placed on the left side of the EyeLink camera to produce a good enough CR to enable high-quality eye tracking (see Fig. 6 part R). The participants of Experiment 5 were the same as in Experiment 4.

To present the visual stimulus, we used a 24-inch ASUS VG248QE monitor (53.0 × 30 cm; 1920 pixels × 1080 pixels (16:9 ratio); refresh rate: 240 Hz) placed at a distance of 74 cm from the eye. The visual stimulus and the instructions to the participant were identical to those in the other experiments.

### Results and discussion

**Fig. 7:**
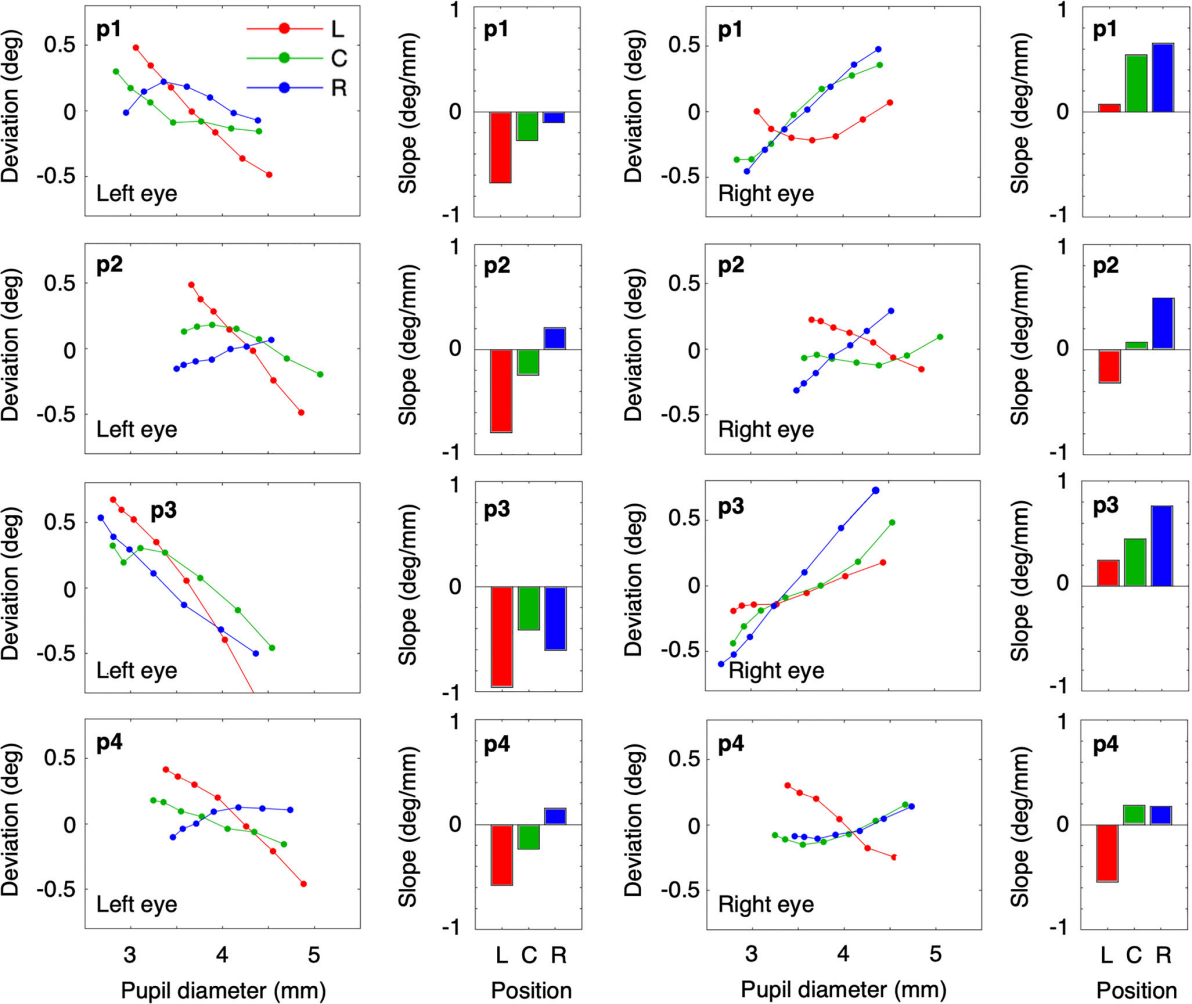
The horizontal component of the PSA and the slope of the horizontal component of the PSA for three different orientations of the EL1000p. *Red* denotes L (left from the participant, seen from the perspective of the participant), *green* denotes C (center, meaning in front of the participant) and *blue* denotes R (right from the participant). Each *row* of panels represents data for one participant. The first panel (*from left to right*) depicts the deviation as a function of pupil size for the left eye; the *second panel* depicts the slopes for the lines of the first panel. Panel 3 and 4 depict the same for the right eye. The slope of the pupil-size artefact denotes the apparent deviation from the gaze direction in degrees per millimeter increase of the pupil diameter

Figure 7 shows the slopes of the pupil-size artefact for the left and right eyes for four participants in the left (L, see Fig. 6), center (C) and right (R) conditions. The data for the four participants look different. However, there is a general pattern in the data. The slopes of the pupil-size artefact are mainly more negative for the left eye and more positive for the right eye.^2^ The data for the left position of the eye tracker (L) most resemble the results obtained with an EL1000p in desktop mode (left eye slopes are negative, right eye slopes are closer to zero). For the center position condition (C), the slopes of the left eye are also less negative than for the leftward condition. However, the pattern more resembles the symmetric pattern (negative slopes in the left eye, positive slopes in the right eye) obtained with the Spectrum (cameras in the center position). For the right condition (R), the slopes of the right eye are more positive than the slopes of the left eye, the slopes of the left eye are closer to zero. We see this pattern as evidence that the pupil-size artefact is dependent on the eye-tracker orientation relative to the participant.

**Fig. 8:**
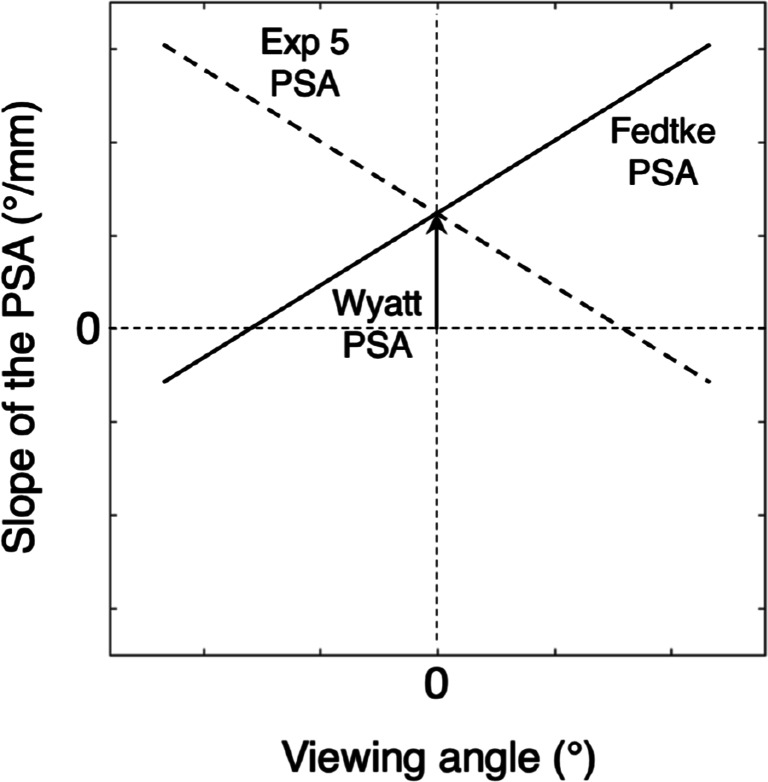
The slope of the PSA is hypothesized to consist of two components (Choe et al., 2016). The first component, which we refer to as the Wyatt PSA, is idiosyncratic and the slope of the PSA may be negative, zero, or positive (Wildenmann and Schaeffel, 2013). It can be estimated by the offset (slope of the PSA when the viewing direction is 0^∘^) and in this figure it is positive. The second component depends on the viewing direction. Here the viewing direction is defined as the angle between the camera axis and orientation of the eye and is positive if the gaze point is right of the camera. The model of Fedtke et al., (2010) predicts the slope of the PSA to increase with viewing angle (*solid line*). Based on the results of Experiment 5, we expect the slope of the PSA to decrease with viewing angle (*dashed line*)

In Experiment 5, we varied the orientation of the eye-tracker camera, while the participants fixated the center of the screen. Therefore, we hypothesize that it is the orientation of the camera axis relative to the participants viewing direction^3^ that affects the slope of the pupil-size artefact (PSA). For both eyes, we found larger (more positive) slopes when the relative angle (the angle between the viewing direction and the eye-tracker-camera axis) was negative (i.e., when the gaze point was located left of the camera) and smaller (more negative) slopes when the relative viewing angle was positive (i.e., when the gaze point was located right of the camera). Figure 8 illustrates this relation (dashed line).

It seems that the model for the pupil-size artefact is more complicated than what Wyatt predicted in 1995 and validated in 2010. The model should at least be able to describe two aspects of the observed pupil-size artefact, namely the original Wyatt part for looking straight ahead and the viewing-direction-related part.

We refer to the first part (the pupil-shape dependent PSA) as the Wyatt PSA. Choe et al., (2016) wrote about the Wyatt PSA: “We reckon that the inter-observer differences were probably due to the idiosyncrasy in pupil shape and to the magnitude of the center shift during constriction (“decentration”) relative to the corneal center”. The Wyatt PSA is the component of the PSA that can be empirically measured when the lines of sight of the eye and the (eye tracker) camera coincide. The slope of the Wyatt PSA may be negative^4^, zero or positive depending on one’s eye physiology (Wildenmann and Schaeffel, 2013).

In order to develop a model to enable us to understand and predict quantitatively the viewing-direction related PSA, we searched the literature for theories predicting a relation between viewing direction and perceived pupil decentration. The first study hypothesizing about a relation between the pupil-size artefact and viewing direction is Choe et al., (2016). They reported differences in the PSA magnitude between the two eyes and related it to the fact that the eye camera of the EL1000 is located on the left side (see figure 1 from Choe et al., 2016). Because it is on the left side, the angle between the camera axis and the line of sight for the left eye is smaller than for the right eye. Choe et al., (2016) wrote on page 57:

“This differential geometry between the eyes probably produced slightly different 2D images of the pupils that, in turn, could have contributed to the different [PSAs] due to viewing-direction-dependent nonlinear distortions that occur when recovering 3D information from 2D images”.

Choe et al., (2016) also provide theoretical substantiation for their observation. They refer to a simulation study by Fedtke et al., (2010) that shows that if the eye is observed from an angle, the perceived pupil is deformed asymmetrically. Fedtke et al., (2010) wrote on page 22364:

“Our three-dimensional model shows that as viewing angle increases, the entrance pupil moves forward, tilts and curves towards the observer’s direction. Moreover, the tangential pupil size narrows and exhibits asymmetric distortions. Consequently, its shape is non-elliptical and its geometric mid-point departs from the optical center. These findings may have implications on the accuracy of peripheral ocular measurements”.

As gaze estimation is based on the determination of the location of the pupil center in the (eye tracker) camera image, gaze estimation could be affected by these asymmetric distortions.

Fedtke et al., (2010) simulated the situation of an observer (person or camera) looking at an eye from the side. In Fedtke’s simulation, the viewing direction of the eye is kept constant and the observer (or camera) viewing angle is varied. Here we apply the model predictions obtained for a situation with a varying observer angle and a fixed eye orientation to a situation resembling that of an eye tracker setup (fixed camera orientation, varying eye orientation). We also want to express the model predictions in the coordinate system used in the current study. To keep it simple, we limit ourselves here to only horizontal manipulations.

**Fig. 9:**
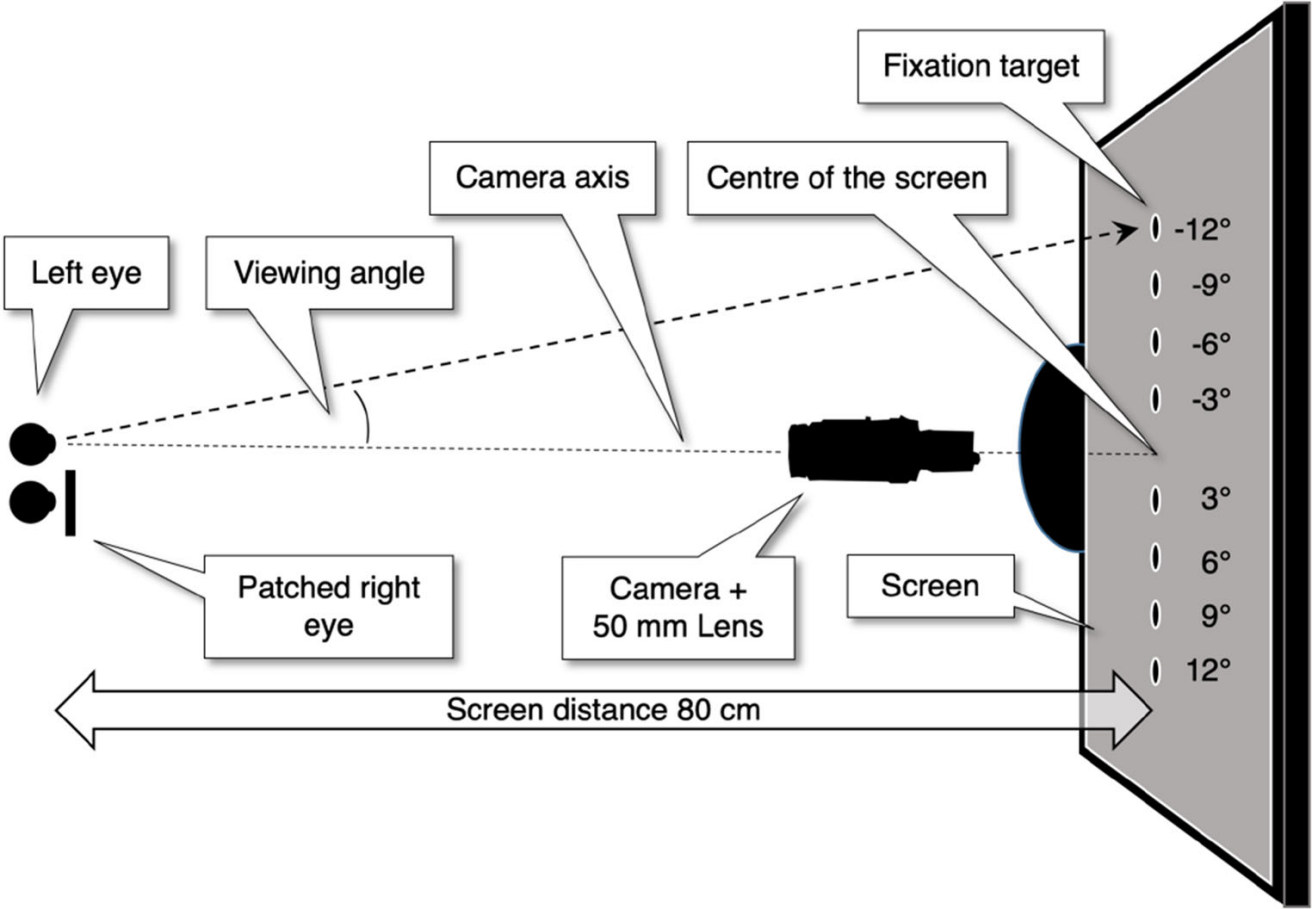
Top view of the setup used in Experiment 6. The camera is located at eye height and placed such that the camera axis and the line of sight coincide if the (occluded) screen center is fixated. Fixation targets left of the camera are denoted with negative viewing angles, targets right of the screen are denoted with positive angles. Viewing is monocular with the left eye, the right eye is patched

We consider the situation consisting of a camera directly in front of the left eye. The left eye is directed at a point located in the horizontal plane (as in Fig. 9). To describe our coordinate system, we take the perspective of the eye and denote eyeball rotations in the rightward direction as positive. For this specific setup, Fedtke et al., (2010) have three predictions for the observed pupil center of the left eye (see Fedtke’s figures 1, 6b and 7).

1. For fixed positive viewing angles, the perceived pupil center moves in the right direction (nasal direction) with increasing pupil size. For positive viewing angles the slope of the PSA is positive.
2. For fixed negative viewing angles, the perceived pupil center moves in the leftward direction (temporal direction) with increasing pupil size. For fixed negative viewing angles the slope of the PSA is negative.
3. The magnitude of the PSA increases with increasing absolute viewing angle.

In summary, the viewing-direction-dependent PSA is in the same direction as the viewing direction and scales with viewing angle. Note that the descriptions of the direction of the apparent pupil center decentration in Fedtke et al., (2010) and Mathur et al., (2013)^5^ seem opposite to our description of their predictions, but are indeed similar, because they consider the direction of the apparent pupil decentration from the perspective of the observer (camera) looking at the eye. We consider it from the perspective of the eye. Figure 8 shows the predictions of Fedtke for the viewing angle-dependent PSA (solid line) and it is contrasted with the prediction based on empirical results from Experiment 5.

## Experiment 6. How does the pupil-size artefact depend on viewing direction?

In Experiment 6, we measured the slope of the PSA versus viewing direction and investigated whether this relation can be described with the Wyatt PSA and the viewing-direction-dependent PSA. To be able to do that, we placed the camera of our FLEX eye tracker in front of the left eye so that it covered the center from the screen (Fig. 9). In separate trials, we measured the slope of the PSA for different viewing directions. According to Fedtke et al., (2010), the slope of the horizontal component of the PSA should increase with viewing angle. Fedtke et al., (2010) predicts the slope to be zero when the line of sight and the camera axis coincide (viewing direction 0^∘^). A nonzero slope for viewing direction of 0^∘^ is interpreted as evidence for the Wyatt PSA (Fig. 8). The evidence for the Wyatt PSA is even stronger if we find different values for the offset across different participants.

### Methods

#### Apparatus

**Fig. 10:**
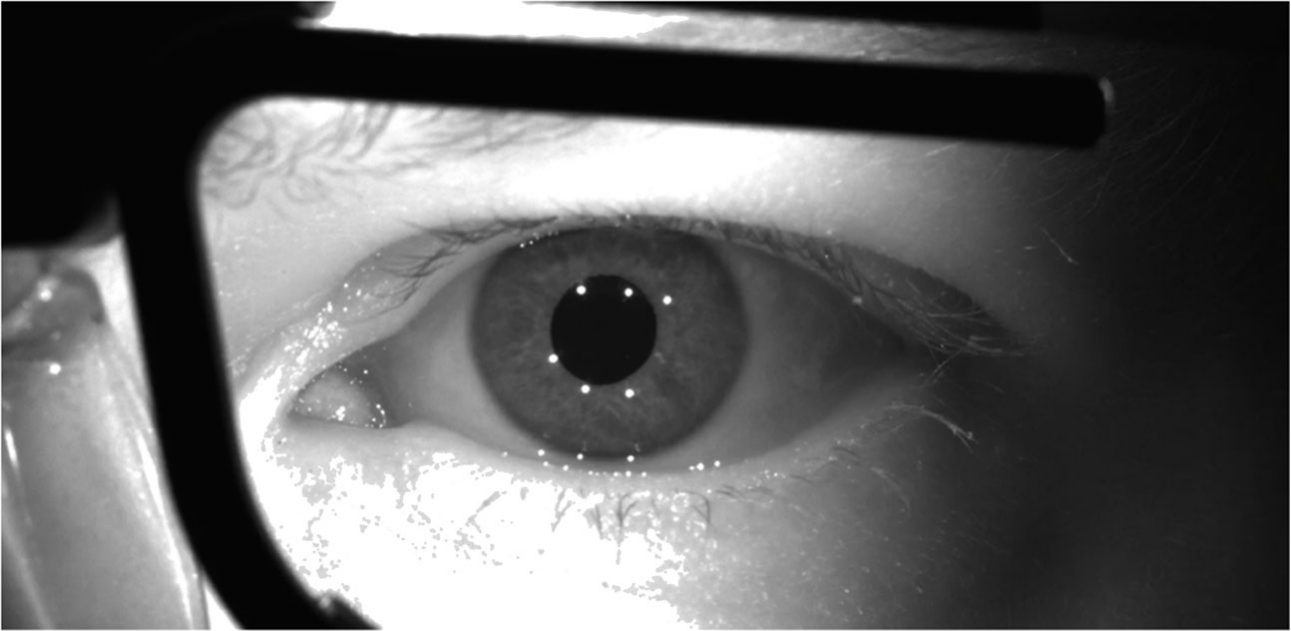
Example of an eye image of the self-built eye tracker (FLEX). The IR illumination is provided by a Tobii Pro glasses 2 wearable eye tracker. The Tobii Pro glasses 2 have six IR illuminators. In the image, the glasses frame and the six corneal reflections are visible. In our image analysis, we used the three lower corneal reflections and the pupil for gaze estimation

We placed a camera (Basler ace acA2500-60um) with a 50-mm lens (AZURE-5022ML12M) and a near-IR long pass filter (MIDOPT LP715-37.5) directly in front of the participant such that the optical axis of the eye and the camera coincide when the participant looks straight ahead (see Fig. 9). Illumination of the eye was delivered by a pair of Tobii Pro Glasses 2. The Tobii has six IR-illuminators producing six corneal reflections in the eye image (Fig. 10). The three lower corneal reflections were used in the image analysis to estimate gaze. Note that the Tobii glasses were only used for illumination of the eye and the production of the CRs. The screen was placed at 80 cm from the left eye. The camera filmed at 150 Hz; Image dimensions were 1216 × 600 pixels; Video was captured at 10-bit resolution with custom software that streamed the recorded frames into an mp4 file using libavcodec (ffmpeg) version 4.2.2 and the libx264 h.264 encoder (preset: veryfast, crf: 17, pixel format: gray10).

#### Eye image analysis

Offline image processing was done at 8-bit resolution. Identification of pupil and corneal reflection centers in the eye images were performed offline using Python 3.7.3 and Open CV 3.4.1. Candidate pupil and CR regions in each video frame were extracted by thresholding (manually set for each video), creating binary images outlining very dark (pupil) and very bright (CR) regions. Each binary blob was tested against size and shape criteria to exclude regions unlikely to belong to a pupil or a CR. The center of mass of the selected pupil and CRs blobs passing this check was computed. The average position of the three lower CRs was used in gaze estimation (Fig. 10). Calibrated gaze data were generated through a ten-point calibration (grey background) where pupil-CR vectors were mapped to calibration target positions on the screen with a second order polynomial. We converted the pupil signal to millimeters by an off-line calibration that consisted of the recording of a measurement tape placed at the position of the left eye to determine the conversion ratio. In this experiment, we did not consider the foreshortening of the pupil due to the angle between the gaze direction and camera axis (maximal 12^∘^). In layman’s terms, if a frontally viewed eye is rotated to the left, the projection of the pupil appears smaller due to the perspective. Here the foreshortening factor is roughly 1/cos(12^∘^), resulting in an underestimation of the real pupil size by 2.2%.

#### Participants, procedure, and stimulus

Four participants (p1, p2, p3, and p14) viewed the screen monocularly with the left eye (the right eye was patched). P1 is the first author, p2 is the last author, p3 is the second author, and p14 is a male naive to the purpose of the experiment. In eight trials, the participants looked at eight different targets. Viewing directions ranged from − 12^∘^ to 12^∘^ with steps of 3^∘^. The 0^∘^ direction was absent because the camera occluded the center of the screen (see Fig. 9). To evoke pupil changes during fixation, we changed the screen from black to white in a sinusoidal manner (f = 0.125 Hz). Presentation time of each trial was 80 s (ten periods).

### Results and discussion

**Fig. 11:**
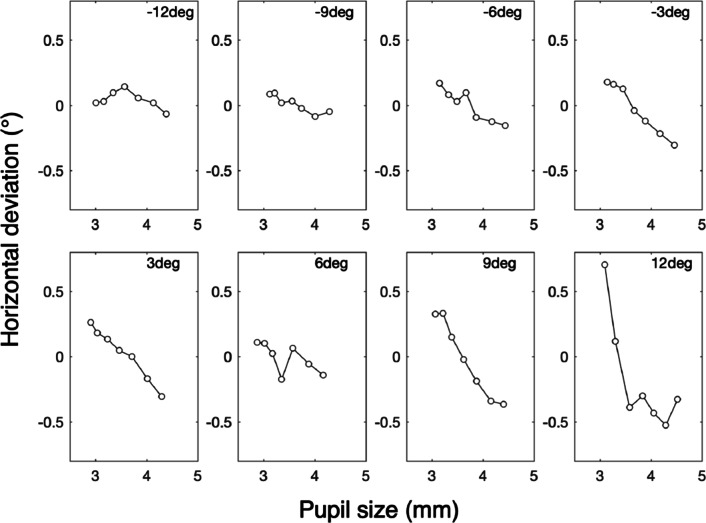
Horizontal deviation of gaze versus pupil size. The eight panels show the PSA for eight different viewing directions, ranging from − 12^∘^ to 12^∘^ for participant p1. For each of the panels, we determined the slope of the PSA by fitting a line through the single points. The slopes are depicted in Fig. 12

**Fig. 12:**
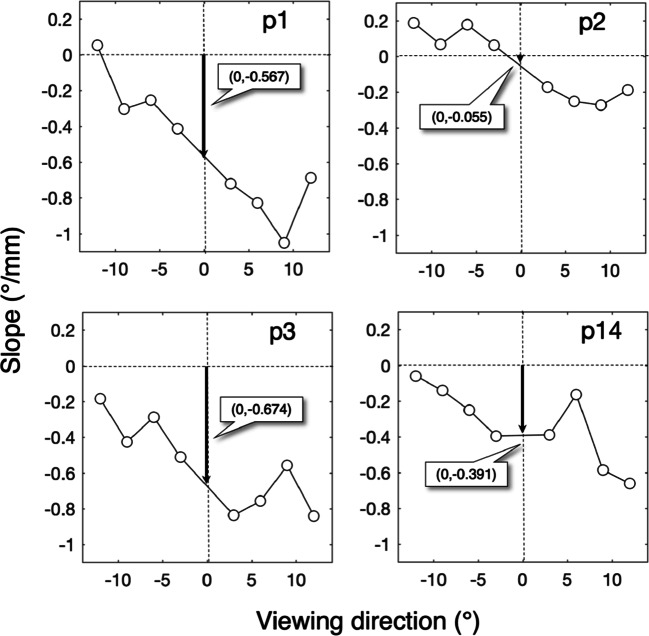
Slope of the PSA versus viewing direction. Each panel shows data for one participant. The *y*-axis denotes the slope of horizontal component of the pupil-size artefact (PSA) in deg/mm. For example, − 0.567^∘^/mm (p1) indicates that for each mm of pupil enlargement, the horizontal component of deviation of gaze is − 0.567^∘^ in the nasal direction (or to the left for the left eye)

Experiment 6 was designed to investigate whether the viewing-direction-dependent slope of the PSA consists of the independent contributions of the Wyatt PSA and the viewing-direction-dependent PSA (see Fig. 8). Figure 11 shows the horizontal component of the PSA for eight different viewing directions for the left eye for participant p1. As viewing direction increases from − 12^∘^ to 12^∘^, the slope of the PSA decreases (becomes more negative). Figure 12 depicts these slopes as a function of viewing direction for four participants. The proportion of data loss (empty samples) per trial ranged from 0.011 to 0.138, with a mean value of 0.0650 and a standard deviation of 0.0291. Each panel contains information about the two hypothesized components of the PSA (Fig. 8). Here, the Wyatt PSA is represented by the offset. Choe et al., (2016) suggested that the Wyatt PSA is represented by the slope of the PSA for viewing direction 0^∘^. The estimated slope of the PSA at 0^∘^ ranges from − 0.674 ^∘^/mm (p3) to − 0.055 ^∘^/mm (p2). For all participants, the slope of the PSA decreases with viewing angle in the same way. This is in line with the result of Experiment 5 in which the slope of the PSA decreased with viewing angle. Our results do not agree with the predictions from the model of Fedtke et al., (2010). The latter model predicts the slope of the PSA to increase with viewing angle (Fig. 8).

## Modeling the role of corneal refraction in PSA

In retrospect, the Fedtke model is probably not the right model to answer our question because it was not developed to predict the PSA, it was developed to: “extend the existing work on peripheral entrance pupil by modeling and assessing the three-dimensional entrance pupil position, shape, and centration as a function of viewing angle and pupil size” (Fedtke et al., 2010, page 22366). Our goal is to propose an anatomy and optics-based model to explain the component of PSA that results from viewing angle variation. Note that this refraction model does not address the component of PSA that results from physiological shift of the pupil center within the eyeball. To be able to do so, we need to model both the eye and the eye tracker. In the next section, we briefly explain 1) the steps by which a pupil-CR eye tracker estimates gaze and 2) how the optics of the eye may cause the viewing-direction-dependent part of the PSA.

We consider the most simple form of a pupil-minus-CR eye tracker setup (such as our FLEX). In this head-fixed set up, the participant looks at a screen in front of her. The eye tracker computes the gaze position on the screen (in pixels, cm’s or degrees). To enable the eye tracker to deliver gaze posi- tions on a screen, the following steps^6^ have to be conducted:

1. Illuminating the eye with one (or more) point IR-light sources.
2. Capturing video from the eye
3. Processing the 2D images of the eye to identify and locate the eye’s corneal reflection (CR) and pupil center within the camera image.
4. Transforming the pupil-CR signal into a gaze position signal in screen coordinates. For this transformation, we used a calibration function that is produced by fitting the p-CR coordinates obtained during fixations to known positions on the screen. Note that we could transform directly from positions on the screen to viewing angle because the head was fixed by a chin- and forehead rest.

### The perceived pupil center and optical corneal refraction

**Fig. 13:**
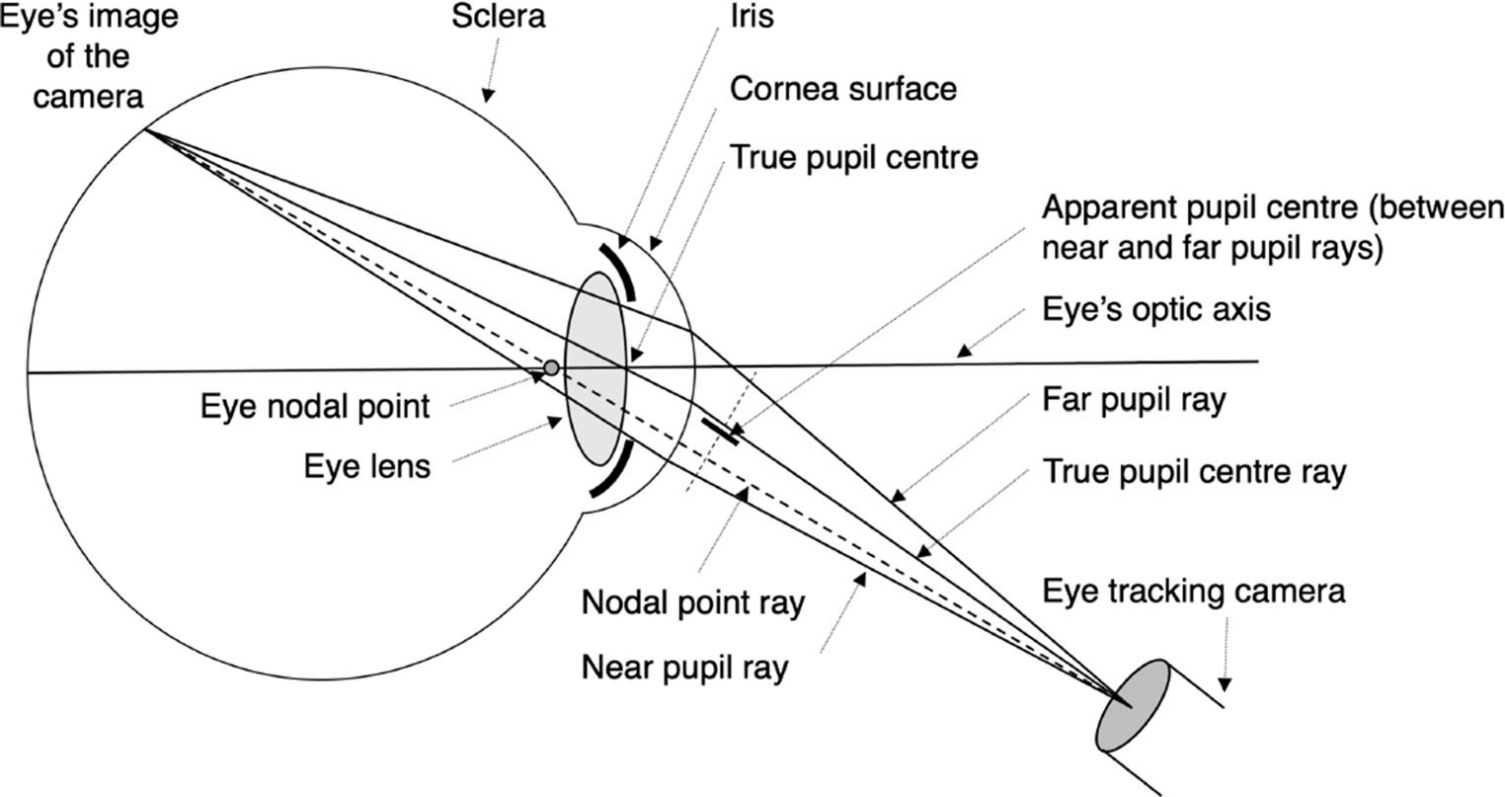
Model of a simplified eye to illustrate the optical PSA. This diagram is exaggerated to illustrate the critical optical PSA effects. The apparent pupil center is modeled by taking the average of the observable far pupil ray and the observable near pupil ray. All these rays, refracted at the corneal surface, travel to the camera. The amount of refraction in each ray depends on a) the shape of the cornea, b) the geometric location of the ray’s origin point behind the corneal surface, and c) the orientation of the camera with respect to the eye’s optic axis. All this geometry affects the shape and location of the pupil image captured by the camera

Optical deformations of the pupil occur because the eye tracker camera views the eye’s pupil through the curved, refracting surface of the eye’s cornea (Fig. 13). To evaluate the effects of corneal refraction on PSA, let us assume that the pupil is perfectly circular and the pupil center is constant and located right on the eye’s optic axis. Even if the eyeball and true pupil center within the eye were to remain perfectly constant, the apparent pupil center, as seen in the eye tracker camera image, shifts as the pupil dilates and constricts. As the true pupil opens and closes concentrically about the pupil center, all the true physical pupil perimeter points move equally toward and away from the pupil center, so the true pupil center is equivalent to the center of all the perimeter points. In the camera image of the pupil, however, the apparent pupil perimeter points move differently because the camera sees each point through a different part of the cornea. Note, if the eye’s optic axis is pointed directly at the camera (viewing angle 0^∘^), all the pupil perimeter points are refracted equally, and there is no optical PSA; but as the eye’s optic axis rotates away from the camera, i.e., as the camera viewing angle increases, the cornea optics refract the various pupil perimeter points with progressively increasing differences, resulting in increasing distortion of the pupil image.

### The gaze signal and optical corneal refraction

How does the corneal-refraction phenomenon affect the gaze (pupil-CR) signal? Note that the apparent position of the corneal reflection (CR), which results only from the reflection of light from the outer surface of the cornea, is not affected by ray refraction through the corneal surface. Thus no refraction-induced PSA results from shifts in the apparent CR location; it all results from shifts in the apparent pupil location.

For an eye with a fixed pupil diameter, both pupil size and shape are deformed differently by refraction of the cornea for each viewing direction. This causes the apparent pupil center to deviate with respect to the unobservable location of the true pupil. However, this deviation is not problematic for gaze estimation because the relation between optically deformed features (e.g., the perceived pupil center in the camera image) and the viewing direction is fixed. The calibration function, if properly chosen, will take care of this fixed relation and the screen coordinates can be mapped automatically onto the p-CR coordinates. The optical deformations are only problematic for the mapping when the pupil size is not equal to the pupil size during calibration. In this case, these optical deformations cause the perceived pupil center position to deviate from the pupil center position used in the calibration, causing apparent deviations in gaze direction. Note that a calibration procedure including pupil size and viewing direction as suggested by Drewes et al., (2014) in principle could prevent the pupil-size artefact.

### The corneal refraction model

Because the detailed optics of the cornea’s refraction and distortion of the pupil-image are highly complicated^7^, we chose to model a simplified eye (Fig. 13). The critical effects of these optical phenomena on pupil-minus-CR eye trackers, however, are shown in this figure, which illustrates the cornea’s refraction of the optical rays that emanate from the pupil-perimeter and reach the camera. The figure shows four important rays from which two are essential for the current modelling:

1. the far pupil ray, a detectable ray that emanates from the pupil perimeter point farthest from the nodal ray,
2. the near pupil ray, a detectable ray that emanates from the pupil perimeter point closest to the nodal ray

These rays, refracted at the corneal surface, travel to the camera. The amount of refraction in each ray depends on a) the shape of the cornea, b) the geometric location of the ray’s origin point behind the corneal surface, and c) the orientation of the camera with respect to the eye’s optic axis (viewing direction). All this geometry affects the shape and location of the pupil image captured by the camera. In pupil-minus-CR eye trackers, the true pupil center ray is not distinguishable within the image; it is typically calculated as the center point of the camera’s pupil image. As illustrated in Fig. 13, the center point between the near and far pupil rays (as seen in the camera image) does not align with the true pupil ray. This misalignment, optically induced by ray refraction at the curved corneal surface, is the source of the optical PSA.

### Simulating PSA

**Fig. 14:**
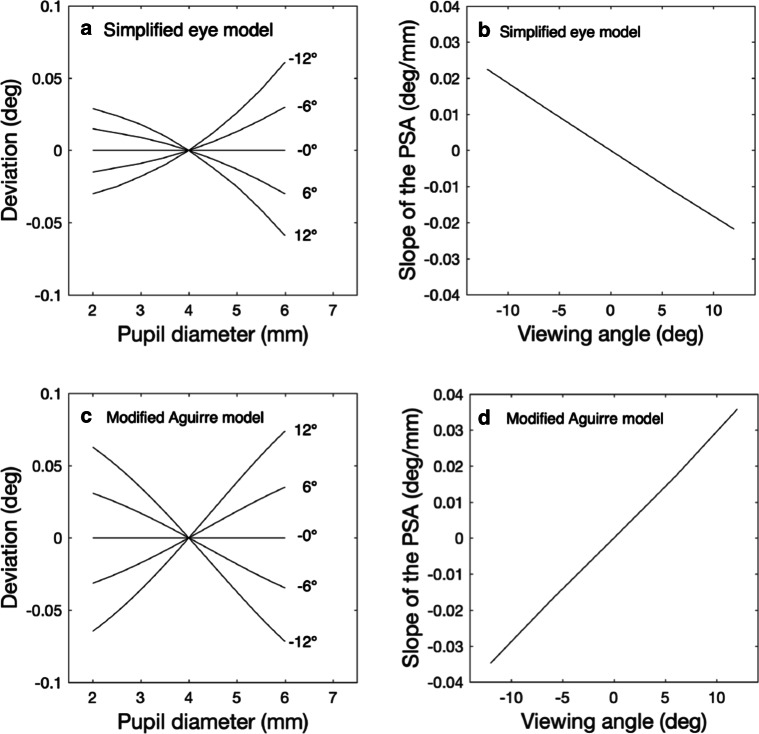
Model simulations of the optical PSA. The top panels (**a** and **b**) contain simulations done with the simplified eye model. Panel **a** depicts deviation versus pupil size for five viewing directions. For negative viewing angles, the slope of the PSA is positive, for positive viewing angles, the slope of the PSA is negative. Panel **b** depicts the slope of the PSA versus viewing angle. The slope of the PSA decreases with increasing viewing angle. The *bottom panels* contain the simulation with the modified Aguirre model (Aguirre, 2019). The magnitude of the PSAs and slopes are comparable to these of the simple eye model, but the effect is in the opposite direction

Our simulation consists of a simplified eye model, a perspective projection on an image plane and a calibration. To simulate the optical PSA, we first setup our model with carefully chosen values for parameters such as eye diameter, corneal surface curvature, position of the nodal point, camera distance and many others (see Appendix A for the details). The simulation started with calibrating the virtual eye tracker. The eye (with a fixed pupil diameter of 4 mm and a camera distance of 53 cm) was rotated from − 20^∘^ to 20^∘^ with steps of 0.001^∘^. For each orientation, we determined the perceived pupil center (the average position of the perspective projections of the far and the near ray in the camera projection plane). From the viewing angles and the corresponding pupil center positions, we constructed a look-up table for converting perceived pupil center position to eye orientation (or viewing angle). To simulate the pupil-size artefact, we set the eye in a fixed orientation and varied the pupil diameter from 2 mm to 6 mm. The look-up table (constructed with a 4-mm pupil) was then used to convert the pupil center position to the apparent orientation of the eye. We computed the deviation in orientation by subtracting the real eye orientation from the apparent eye orientation. This procedure was repeated for five viewing angles (− 12^∘^, − 6^∘^, 0^∘^, 6^∘^ and 12^∘^). Figure 14a shows the gaze deviation as function of pupil diameter for these five viewing angles. The slope of the PSA is positive for negative viewing angles. The slope of the PSA becomes more negative for increasing (more positive) viewing angles (Fig. 14b). Concerning the direction of the PSA, the optical component of the PSA is in agreement with the empirical results from Experiments 5 and 6. The magnitude, however, is much smaller (a factor of 10 to 40, for comparison see Fig. 11). We did not expect this and as a sanity check for our model, we also implemented a modified version of the eye model from Aguirre (2019) in our simulation. We choose the eye model from Aguirre because he made the matlab code of his model available. To make comparison easier, we removed two features from the original Aguirre model. Firstly, we removed the rotation of the apex of the corneal ellipsoid of 2.5^∘^ towards the visual axis of the eye. Secondly, we removed the pupil center decentration with increasing pupil size. Our motivation is that the rotated apex of the corneal ellipsoid only produces an offset in the viewing angle. The motivation for the removal of the pupil center decentration is that this would in principle produce the Wyatt PSA. At this stage, we are not interested in that because we only want to model the optical PSA. Figure 14C and D depict the simulations with the modified Aguirre eye model. The direction of the PSA in the modified Aguirre model is opposite to ours, resulting in a slope of the PSA that increases with viewing angle. The magnitude of the PSA produced by the modified Aguirre eye model is small and comparable to the PSA produced by our simplified eye model.

Although we expect that the viewing-direction component is in fact of optical origin, we could not corroborate this using a simplified eye model and the anatomically detailed modified Aguirre model. The empirical viewing-direction-related PSA has a magnitude that is 10 to 40 times higher than the modelled viewing-direction-related PSA. We have no explanation for the discrepancy between the empirical results and the model predictions.

## General discussion

### Summary of results

In this article, we have asked three questions. 1) How stable is the PSA over time and 2) does the PSA depend on properties of the eye tracker setup and 3) does the PSA depend on viewing direction? By investigating the PSA in the light of these three questions we obtained the following results:

We repeatedly measured the PSA 1) within an hour, 2) on a daily basis and 3) with an interval of a year. The results show that the PSA is very stable over time but may differ between participants (as in Wildenmann and Schaeffel, 2013; Drewes et al., 2014; Choe et al., 2016; Hooge et al., 2019). We also found the PSA to be different and asymmetric between the left and the right eyes for all our participants. In the literature there are reports of the direction of the horizontal component of the PSA to be symmetrical between the eyes (e.g., Wildenmann & Schaeffel, 2013). Others (e.g., Drewes et al., 2014; Choe et al., 2016) report the PSAs having different magnitudes in the left and the right eye. We asked whether the obtained PSA depended on the eye tracker used (as suggested by Choe et al., 2016). First, we compared measurements from the Spectrum with these of the EL1000p. We found that the PSA in the Spectrum is symmetrical between the two eyes. The PSA in the EL1000p is asymmetrical. In the EL1000p the slope of the PSA in the left eye is smaller (more negative) than the PSA in the right eye. Unsurprisingly, the largest PSA effects in the literature (up to 5^∘^) were found by Drewes et al., (2014) with the asymmetric EL1000 set up.

The previous comparison between the PSA from an eye tracker with the camera on the left side (EL1000p) and one with two frontally placed cameras (Spectrum) prompted us to investigate the PSA as a function of the camera orientation. We hypothesized that the relative angle between the camera axis and the participants’ viewing direction affects the magnitude of the PSA in a systematic way. We placed the EyeLink in three orientations (18^∘^ to the left, frontally (0^∘^) and 18^∘^ to the right). For the frontal orientation of the EL1000p we obtained similar results as for the Spectrum, and the left and rightward rotated EyeLink produced similar but a mirrored PSA, suggesting that the angle between the viewing direction and the camera axis affects the PSA.

Choe et al., (2016) suggested that the PSA consists of an idiosyncratic component (that can be observed if the camera axis and the optical axis of the eye coincide) and an optical component (related to the viewing angle with respect to the eye tracker camera). To test the hypothesis of Choe et al., (2016), we investigated how the horizontal component of the PSA depends on the angle between the camera axis and the viewing direction. We used the self-built FLEX eye tracker because it allows us to film the eye frontally (when viewing direction coincides with the optical axis of the camera). The PSA was non-zero for viewing direction 0^∘^ (evidence for the idiosyncratic PSA) and depended on the viewing direction. The slope of the PSA decreased (became more negative) with increasing viewing angle. This result is consistent with the results of the experiment in which we rotated the eye tracker to manipulate the angle between the viewing direction and the camera axis.

The important question then became whether the viewing-direction-dependent PSA is caused by the optics of the cornea. To investigate this, we built a one-dimensional model to simulate the effects of the corneal optics. The direction of the simulated PSAs corresponded to our empirical results, however the magnitude of the simulated PSAs is far too low, a discrepancy we currently do not understand. Based on the simplified eye model, we cannot claim that the viewing-direction-dependent component of the PSA is caused by the optics of the cornea.

### Implications for eye tracking

The results from the present study have a number of implications for eye tracking. The fact that the PSA is very stable over time makes it worth investing in a solution that algorithmically accommodates the optical PSA phenomenon. A calibration method as suggested by Drewes et al., (2014) would be a good candidate. However, their method has the drawback 1) that it has to be implemented by the experimenter and 2) that it takes more time to calibrate compared to a conventional calibration. A method that delivers the same mapping function as proposed by Drewes et al., (2014) and at the same time asks less investments from the experimenters and consumes less time from the participants, would be preferable. We encourage researchers in the eye tracking field to develop such a method and eye tracker manufacturers to implement them in their products. Eyegaze Inc. provides accommodation for optical PSA in its Eyegaze Edge® eye trackers.

Is the PSA a factor to take into account in experimentation? This topic is already discussed in Hooge et al., (2019). They claim that extreme lighting conditions may occur during normal experimenting with eye trackers. As examples, they give driving a car in the shadow and the sun and watching a TV commercial in which the lighting conditions vary from very light to dark. Furthermore, environments such as virtual reality set ups may also provide sufficiently large variations in lighting conditions that substantial PSA may occur. However, we do not want our paper to appear alarmist. Our aim is instead to make researchers aware of the potential significant influence of pupil size and the resulting PSA on eye tracking data quality and thereby spur researchers to have a critical look into pupil size. We advise researchers to inspect the range of pupil sizes occurring during their experiment, to be able to decide whether the PSA may be a factor harming their data quality in the context of their experiment.

The occurrence of the PSA decreases accuracy and thus data quality. What can we do to prevent or minimize the PSA without using an online (Drewes et al., 2014) or offline method (Choe et al., 2016)? Here we present some scenarios

1. Using high light levels during calibration and testing to induce a small pupil that hardly changes size (Hooge et al., 2019). However, it is an empirical question whether it is possible to prevent temporarily increased pupil size due to arousal.
2. Selecting participants on the basis of the absence of the idiosyncratic pupil-size artefact (Hooge et al., 2019). Such candidates can be found among the elderly because pupil size decreases with age (Birren et al., 1950).
3. Using equiluminant color stimuli and equiluminant color calibration screens (Hooge et al., 2019). However, equiluminant stimuli cannot prevent that the arousal state and thus pupil size changes during the experiment. We also do not consider this an easy to apply scenario, because an equiluminancy calibration procedure would have to be run for each participant and each color pair (Anstis and Cavanagh, 1983).
4. To decrease the PSA in general one should use an eye tracker that films the eyes frontally (or by means of a hot mirror) to minimize the average relative viewing angle (angle between the camera’s optical axis and the optical axis of the eye). Note that we do not advise to minimize the viewing angle in general. This is of course relevant when using the EyeLink 1000 family eye trackers in desktop mode. The eye tracker can be rotated so that the camera films the face frontally, however, we do not know whether operating the EL1000 frontally has disadvantages. We can imagine that the illuminator position and orientation become suboptimal.

## Conclusions

The pupil-size artefact is very stable over a long period (at least a year). As proposed by Choe et al., (2016), the pupil-size artefact can be described with two components. First, the pupil center physiologically moves around within the eyeball as the pupillary muscles dilate and constrict (Wyatt, 1995). This pupil decentration behavior, while predictably stable within a given eye, varies between eyes, making it difficult to accommodate without individual calibration. The second component of the PSA depends on the relative orientation of the participants’ eye to the eye-tracker camera axis. Therefore, the pupil-size artefact may differ between different eye-tracking set ups due to differences in the geometry. From the simulations we conclude that the viewing-direction-related PSA cannot be explained by refraction in the cornea only.

## Figures and Tables

**Fig. 15:**
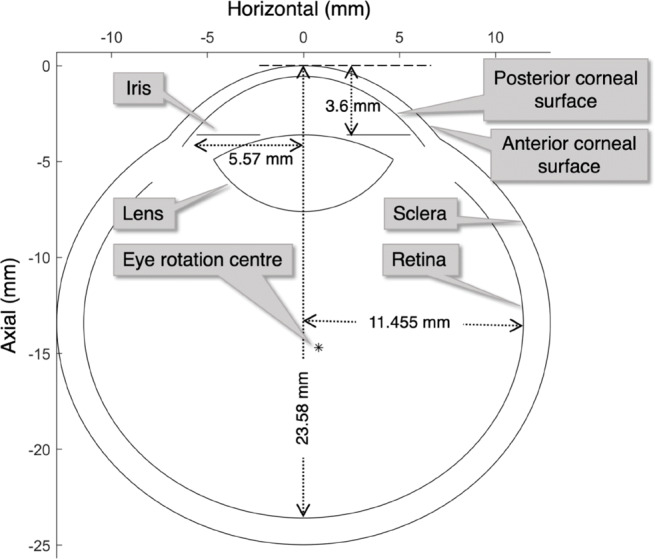
The simplified eye model. More values for the parameters and dimensions can be found in Table 1

## Appendix A

Figure 15 contains a schematic drawing of the eye model used. All the parameters can be found in Table 1.

**Table 1:** Simplified eye model (see Fig. 15) used in this paper, along with values used for its parameters

Parameter	Horizontal (mm)	Axial (mm)	Source	Note
Dimensions				
Eye rotation center	0.79	− 14.7	AG	
Cornea anterior radii	10.43	14.26	AG	
Cornea posterior radii	9.3027	13.7716	AG	
Cornea posterior distance	0	0.55	AG	location of apex of posterior corneal surface
Pupil distance	0	3.6	DR	location of pupil plane
Iris outer radius	5.57	0	AG	
Retina radii	11.455	10.148	AG	
Sclera radii	12.855	11.548	AG & YS	
Axial length	0	23.58	AG	location of back of retina
Lens front radii	9.2	9.2	YS	
Lens front distance	0	3.6	YS	location of front lens surface
Lens back radii	5.4	5.4	YS	
Lens back distance	0	7.6	YS	location of back lens surface
Camera distance	0	530		not shown in Fig. 15
Refractive indices				
Parameter	Index		Source	Note
Cornea	1.3696		AG	
Aqueous	1.3297		AG	
Lens	1.4309		AG	1) for 40-year-old eye, 2) ignoring lens capsule
Vitreous	1.33		AG	

Underlined parameters affect the simulated PSA, other parameters are included only to complete the eye model. Refractive indices are computed using the values in Aguirre (2019), computed for light with a wavelength of 890 nm. Parameter sources: Aguirre (2019), Young and Sheena (1975), and Drexler et al., (1997)

## Footnotes

1 The geometry of the whole setup is defined as the relative and absolute positions and orientations of the eye, the eye-tracker camera, and the IR illuminator.

2 In this experiment, we did not consider the foreshortening of the pupil due to the angle between the gaze direction and camera axis (maximal 18^∘^). The foreshortening factor is roughly 1/cos(18^∘^), resulting in an overestimation of the slopes by 5.1%

3 To describe directions (see Fig. 9), we will use the term viewing direction for the participants looking direction. For description of the orientation of the eye tracker we will refer to the direction of the eye-tracker camera axis. We will use the term viewing angle to describe the angle between the viewing direction and the center of the screen. Fixations on the right side of the screen will be denoted as positive.

4 A left eye’s contracting pupil leads to gaze deviating nasally

5 Mathur et al., (2013) wrote: “It undergoes asymmetric changes in shape, and its center no longer coincides with the on-axis center, moving in the opposite direction to that from which the pupil is viewed”

6 Note that some pupil-minus-CR eye trackers perform some of the steps in a different way. In the calibration function, we implicitly consider eye physics. In other eye trackers (e.g., remote eye trackers), the physics may be dealt with in a more explicit way (e.g., in the form of geometrical calculations).

7 For instance, the physical pupil perimeter rides along the curved anterior surface of the eye’s lens, so the distance between the eye’s optical nodal point and pupil plane—which is a key parameter in the pupil-minus-CR method’s gain coefficient—varies with pupil diameter. The corneal surface is not a perfect sphere. It flattens toward the edges and may exhibit significant astigmatism.

## References

[CR1] Aguirre GK (2019). A model of the entrance pupil of the human eye. Scientific Reports.

[CR2] Anstis, S., & Cavanagh, P. (1983). A minimum motion technique for judging equiluminance. In J Mollon, & L Sharpe (Eds.) *Color Vision: Physiology and Psychophysics* (pp. 155–166). London: Academic.

[CR3] Barsingerhorn AD, Boonstra FN, Goossens J (2018). Development and validation of a high-speed stereoscopic eyetracker. Behavior Research Methods, Instruments.

[CR4] Birren JE, Casperson RC, Botwinick J (1950). Age changes in pupil size. Journal of Gerontology.

[CR5] Choe KW, Blake R, Lee SH (2016). Pupil size dynamics during fixation impact the accuracy and precision of video-based gaze estimation. Vision Research.

[CR6] Cleveland, D. (2003). Method and system for accommodating pupil non-concentricity in eyetracker systems. http://www.google.com/patents/US20030086057

[CR7] Drewes, J., Masson, G. S., & Montagnini, A. (2012). Shifts in reported gaze position due to changes in pupil size: ground truth and compensation. In *Proceedings of the Symposium on Eye Tracking Research and Applications. ETRA 2012* (pp. 209–212). New York: ACM.

[CR8] Drewes, J., Zhu, W., Hu, Y., & Hu, X. (2014). Smaller is better: Drift in gaze measurements due to pupil dynamics. *PloS One, 9*. 10.1371/journal.pone.0111197 PMC4206464 25338168

[CR9] Drexler W, Baumgartner A, Findl O (1997). Submicrometer precision biometry of the anterior segment of the human eye. Invest Ophthalmol Vis Sci.

[CR10] Fedtke C, Manns F, Ho A (2010). The entrance pupil of the human eye: A three-dimensional model as a function of viewing angle. Optics Express.

[CR11] Hooge ITC, Camps G (2013). Scan path entropy and arrow plots: Capturing scanning behavior of multiple observers. Frontiers in Psychology.

[CR12] Hooge ITC, Hessels RS, Nyström M (2019). Do pupil-based binocular video eye trackers reliably measure vergence?. Vision Research.

[CR13] Jaschinski, W. (2016). Pupil size affects measures of eye position in video eye tracking: implications for recording vergence accuracy. *Journal of Eye Movement Research, 9*.

[CR14] Kristek A (1965). The physiological pupillogram of the human eye. Czechoslovakian J. Ophthalmol..

[CR15] Mathur A, Gehrmann J, Atchison DA (2013). Pupil shape as viewed along the horizontal visual field. Journal of Vision.

[CR16] Merchant J, Morrissette R, Porterfield JL (1974). Remote Measurement of Eye Direction Allowing Subject Motion Over One Cubic Foot of Space. IEEE transactions on Biomedical Engineering.

[CR17] Morimoto CH, Koons D, Amir A, Flickner M (2000). Pupil detection and tracking using multiple light sources. Image and Vision Computing.

[CR18] Mulligan JB (1997). Image processing for improved eye-tracking accuracy. Behavior Research Methods, Instruments, & Computers.

[CR19] Peirce JW (2007). PsychoPy - Psychophysics software in python. Journal of Neuroscience Methods.

[CR20] Peirce, J. W. (2008). Generating stimuli for neuroscience using PsychoPy. *Frontiers in Neuroinformatics, 2*(10). 10.3389/neuro.11.010.2008 PMC2636899 19198666

[CR21] Walsh G (1988). The effect of mydriasis on the pupillary centration of the human eye. Physiological Optics.

[CR22] Wildenmann U, Schaeffel F (2013). Variations of pupil centration and their effects on video eye tracking. Ophthalmic and Physiological Optics.

[CR23] Wyatt HJ (1995). The form of the human pupil. Vision Research.

[CR24] Wyatt HJ (2010). The human pupil and the use of video-based eyetrackers. Vision Research.

[CR25] Young LR, Sheena D (1975). Survey of eye movement recording techniques. behavior research methods and instrumentation. Behavior Research Methods and Instrumentation.

